# Automated synthesis of radiopharmaceuticals for positron emission tomography: an apparatus for labelling with [^11^C] methyl iodide (MIASA)

**DOI:** 10.1155/S1463924694000271

**Published:** 1994

**Authors:** D. G. Cork, H. Yamato, K. Yajima, N. Hayashi, T. Sugawara, S. Kato

**Affiliations:** 1 Institute for Biofunctional Research c/o National Cardiovascular Center Fujishirodai Suita Osaka 565 Japan; 2 Molecular Chemistry Laboratory Takeda Chemical Industries Ltd Todogawa-ku Osaka 532 Japan; 3 Molecular Chemistry Laboratory Takeda Chemical Industries Ltd Yodogawa-ku Osaka 532 Japan; 4 Medical Chemistry Discovery Research Yoshitomi Pharmaceutical Industries Ltd 955 Koiwai Yoshitomi-cho Chikuhjo-gun Fukuoka 871 Japan; 5 Ogiwaradai-nishi 3-chome 184 Hyogo Kawanishi 666 Japan

## Abstract

A fully automated apparatus for the routine synthesis and formulation of short-lived ^11^C (t_1/2_ = 20 min) labelled radiopharmaceuticals for positron emission tomography (PET) has been developed. [^11^C]Carbon dioxide is converted to [^11^C]methyl iodide, which can be used to label a wide variety of substrates by methylation at C, N, O, or S electron rich centres. The apparatus, MIASA (methyl iodide automated synthesis apparatus), was designed to operate as part of an automated labelling system in a shielded ‘hot’ laboratory. The apparatus was designed without the size constraints of typical instrumentation used in hot cells, although it is compact where necessary. Ample use of indicators and sensors, together with compact design of the reaction flasks for small dead space and efficient evaporation, led to good reliability and performance. The design of the hardware and software is described in this paper, together with a preparation of 3-N-[^11^C]methylspiperone as a sterile injectable solution in physiological saline.


Journal of Automatic Chemistry, Vol. 16, No. 6 (November–December 1994), pp. 219–230

### Automated synthesis of radiopharmaceuticals for positron emission tomography: an apparatus for labelling with [$^{11}$C]methyl iodide (MIASA)

**D. G. Cork*†, H. Yamato‡, K. Yajima, N. Hayashi§**
*Institute for Biofunctional Research, c/o National Cardiovascular Center, Fujishirodai, Suita, Osaka 565, Japan*

**T. Sugawara and S. Kato**
*Molecular Chemistry Laboratory, Takeda Chemical Industries Ltd, Yodogawa-ku, Osaka 532, Japan*

*A fully automated apparatus for the routine synthesis and formulation of short-lived $^{11}C$ ($t_{1/2} = 20$ min) labelled radiopharmaceuticals for positron emission tomography (PET) has been developed. [$^{11}$C]Carbon dioxide is converted to [$^{11}$C]methyl iodide, which can be used to label a wide variety of substrates by methylation at C, N, O, or S electron rich centres. The apparatus, MIASA (methyl iodide automated synthesis apparatus), was designed to operate as part of an automated labelling system in a shielded 'hot' laboratory. The apparatus was designed without the size constraints of typical instrumentation used in hot cells, although it is compact where necessary. Ample use of indicators and sensors, together with compact design of the reaction flasks for small dead space and efficient evaporation, led to good reliability and performance. The design of the hardware and software is described in this paper, together with a preparation of 3-N-[$^{11}$C]methylspiperone as a sterile injectable solution in physiological saline.*

### Introduction

Positron emission tomography (PET) has developed into a unique tool for obtaining quantitative physiological images of biofunctions. A variety of applications are being pursued—these are based on the fact that positron emitters, such as $^{11}$C, $^{13}$N or $^{15}$O, can be incorporated into almost any biologically active tracer without altering the chemical behaviour.

The labelling of pharmaceuticals with the positron emitter $^{11}$C for use in PET studies is frequently accomplished using [$^{11}$C]methyl iodide as a labelling agent. Figure 1 shows the synthetic scheme used. The short half life of $^{11}$C (20·4 min) means it is essential to synthesize the radiopharmaceuticals regularly and consistently on site. It is usually necessary to start with relatively high levels of radioactivity to obtain useful amounts of the required products. So automation of the synthesis apparatuses, for example 3–6, within a radiation-shielded facility is important for safe, reliable and efficient production of radiopharmaceuticals for PET.

A number of remote controlled and semi- or fully automated systems have been developed for labelling [1–5]. However, although these systems have begun to address the need for producing labelled compounds, PET has begun to move out of the research arena to become a routine clinical tool and this is putting ever greater demands on the automated apparatus. A reliable system that can reproducibly deliver a variety of radiopharmaceuticals on a routine, repetitive basis would be desirable. The commercial synthesis instruments on the market are generally limited in their scope; need manual washing before re-use; and/or require the attendance of specialist operators or maintenance personnel.

A total system for routine production of PET radiopharmaceuticals is being developed at the Institute for Biofunctional Research (IBR). This paper reports on the design and construction of an apparatus for producing radiopharmaceuticals by labelling with [$^{11}$C]methyl iodide—MIASA (methyl iodide automated synthesis apparatus)—and describes its application to synthesis of 3-*N*-[$^{11}$C]methylspiperone as a sterile injectable solution in physiological saline.

### General features

Figure 2 shows the general layout of the facilities at IBR which were designed for the development of a total production system from the cyclotron to the PET camera. The production of $^{11}$C is accomplished by the nuclear reaction of cyclotron-accelerated protons with nitrogen gas, $^{14}N(p, \alpha)^{11}C$, in a target chamber. A Sumitomo Heavy Industries HM-18 cyclotron is used for this. Pico mole quantities of $^{11}$C undergo rapid oxidation to [$^{11}$C]carbon dioxide in the target chamber or by passage over a CuO catalyst. Incorporation of the positron emitters into the radiopharmaceuticals takes place in the hot laboratory, which is designed to contain several fully automated synthesis instruments under computer control. Communication with a network of OPTOMUX (Opto 22, USA) modules allows flexible control and monitoring of all devices [4, 6]. After quality assurance procedures have been completed, the sterile product is passed through to the PET camera room.

* *Correspondence to D. G. Cork.*
† *Present address: Molecular Chemistry Laboratory, Takeda Chemical Industries Ltd, Yodogawa-ku, Osaka 532, Japan.*
‡ *Present address: Medical Chemistry Discovery Research, Yoshitomi Pharmaceutical Industries Ltd, 955 Koiwai, Yoshitomi-cho Chikuhjo-gun, Fukuoka 871, Japan.*
§ *Present address: Ogiwaradai-nishi 3-chome 184, Kawanishi, Hyogo 666, Japan.*

0142-0453/94 $10 00 © 1994 Taylor & Francis Ltd.

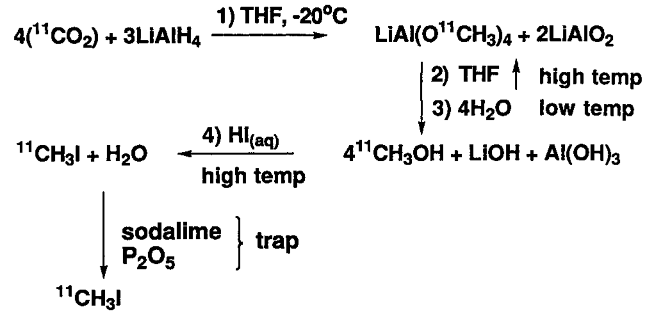

*Figure 1. Scheme for the synthesis of [$^{11}$C]methyl iodide.*

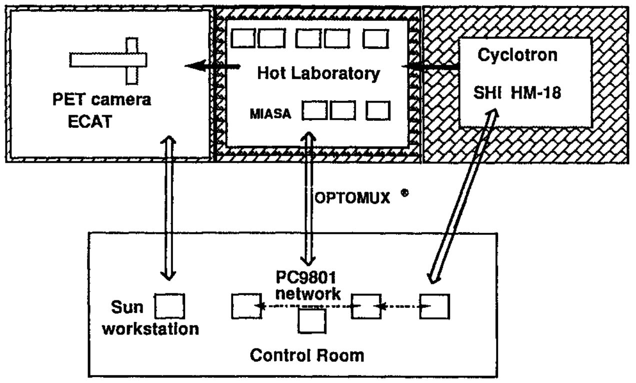

*Figure 2. Layout of the PET radiopharmaceutical production laboratories.*

### Computer control system

### *Computer and interface hardware*

The apparatus is controlled by a personal computer (NEC 9800 series) with standard monitor and printing peripherals. The computer communicates with an OPTOMUX interface unit via a plug in RS422 adaptor card and an interface board (FCB485, Asahi Electronics, Japan). Six OPTOMUX digital and two analogue brain boards, each with 16 I/O channels, are used. Five digital output boards give 80 switches for operating valves and relays, and one digital input board reads the status of 16 indicator lamps that are used for monitoring photosensors, position and level sensors. The analogue boards are for reading status information from the system, including temperature, pressure, radioactivity, pH, UV absorbance, and writing information to set temperature and gas flow rates.

### *Computer software*

The computer software for controlling the apparatus was developed using a control software development tool called 'Hyakuninriki' (Asahi Electronics, Japan) operating under MS-DOS. The program consists of a series of connected control blocks, with the flow depending on the control logic sequence. More than 100 control blocks of 16 different types are used, and each block is programmed to perform a function, such as controlling a switching sequence or reading the status of sensors. An example of the layout of blocks and their connections for the control of the HPLC injection procedure is shown in figure 3. Figure 4 is a flowchart of the same procedure and a more detailed explanation is given below for the injection procedure during the synthesis of 3-*N*-[$^{11}$C]methylspiperone.

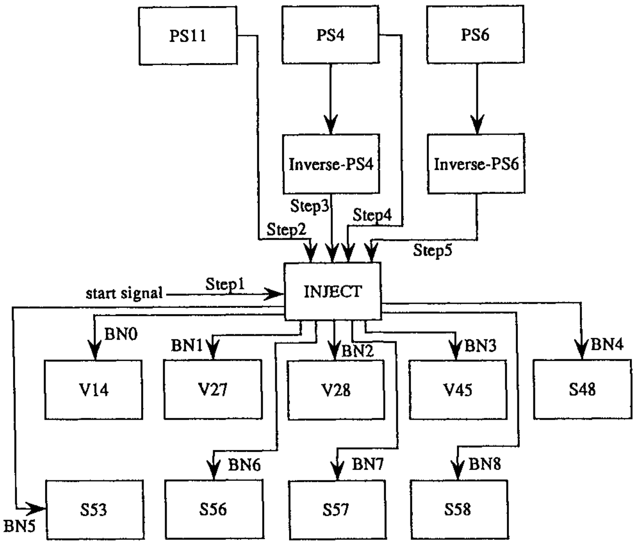

*Figure 3. Control blocks for HPLC injection: PS = photosensor; BN = bit number.*

The flowchart for checking the integrity of the apparatus, the diagnostic check, is shown in figure 5. The status of the apparatus is confirmed to be ready for synthesis by intrachecking photosensors, micro-switch sensors, rotary valve positions and performing leak tests, thus helping to maintain reproducible and safe operation of the apparatus. A typical program flowchart for a synthesis and formulation procedure is shown in figure 6.

### The synthesis apparatus

### *General*

As the apparatus did not need to be operated in the confines of a hot cell, it was possible to organize the hardware for easy maintenance and for good reliability and reproducibility. The reaction unit was kept compact in order to minimize dead space in the two reaction flasks (F1 and F2) and flow lines, which can lead to dilution and losses of the $^{11}$C-labelling agents, but the peripheral supply and service units were laid so that they were easily accessible. The organization of the units on the two racks (main and supply, 60 × 40 × 180 cm) is shown in figure 7, and a schematic diagram of the apparatus is shown in figure 8. The general appearance of the apparatus and the reaction unit are shown in figures 9 and 10. Table 1 lists the I/O connections of the MIASA apparatus.

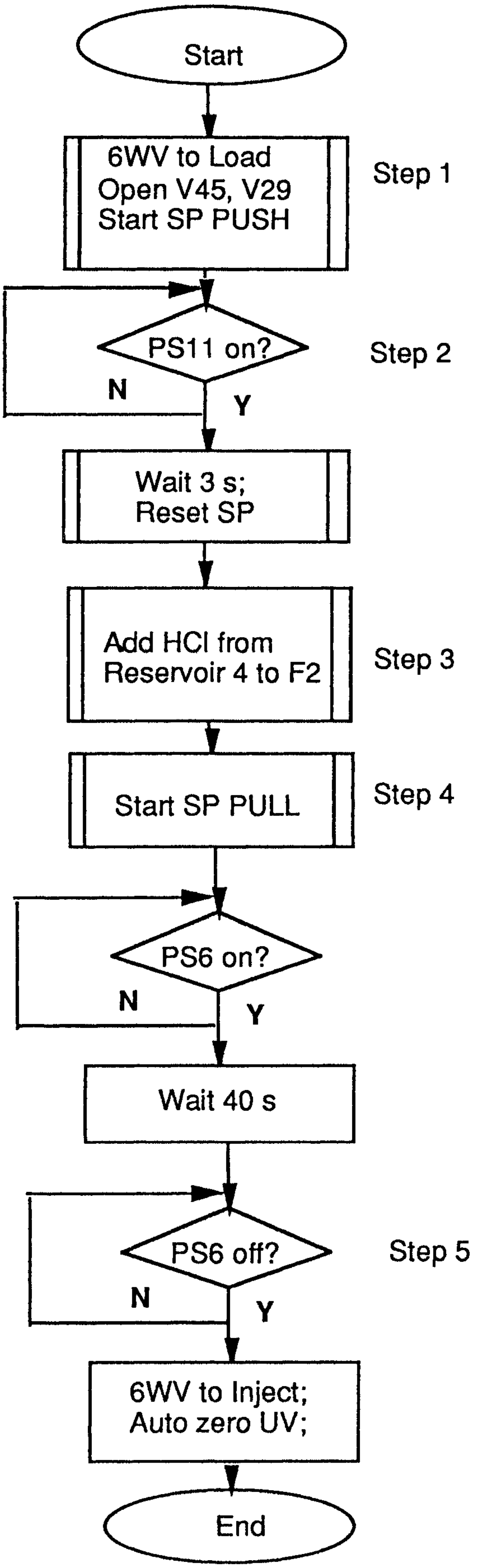

*Figure 4. The HPLC injection procedure: 6WV = six way rotary valve; SP = syringe pump; F2 = reaction flask #2.*

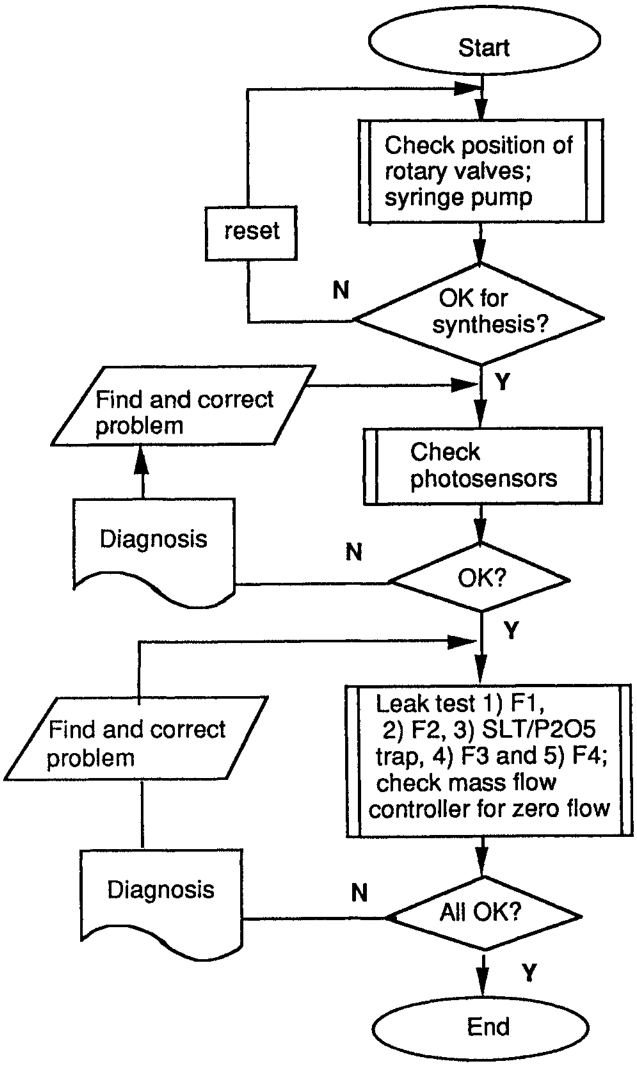

*Figure 5. The diagnostic check sequence.*

*Layout of the main rack*

*Electronics unit*

Control boxes for the magnetic stirrers and pH meter (NPH-10D, Nissin), 24V and 12V DC power supplies and cable connectors are located on the top shelf of the main rack. Temperature control of three reaction flasks (F1–F3) is performed by three thermostat controllers (E5 series, Omron), each with a variable voltage thyristor. Two of the flasks, F1 (methyl iodide synthesis) and F3 (formulation), use local control with a preset temperature value and simple on/off capacity, but the temperature of flask F2 (labelling) may freely be set remotely from the computer during synthesis. This feature enables the temperature to be set at a low value for trapping methyl iodide and then increased for rapid labelling.

The control-boxes for a syringe-pump motor (SMPC-001, Oriental Motor) and a liquid-level sensor on flask F4 (AL-66r, Nissin), and eight 37-pin cable connectors to the I/O indicator–auto/manual switch box are also located on this shelf.

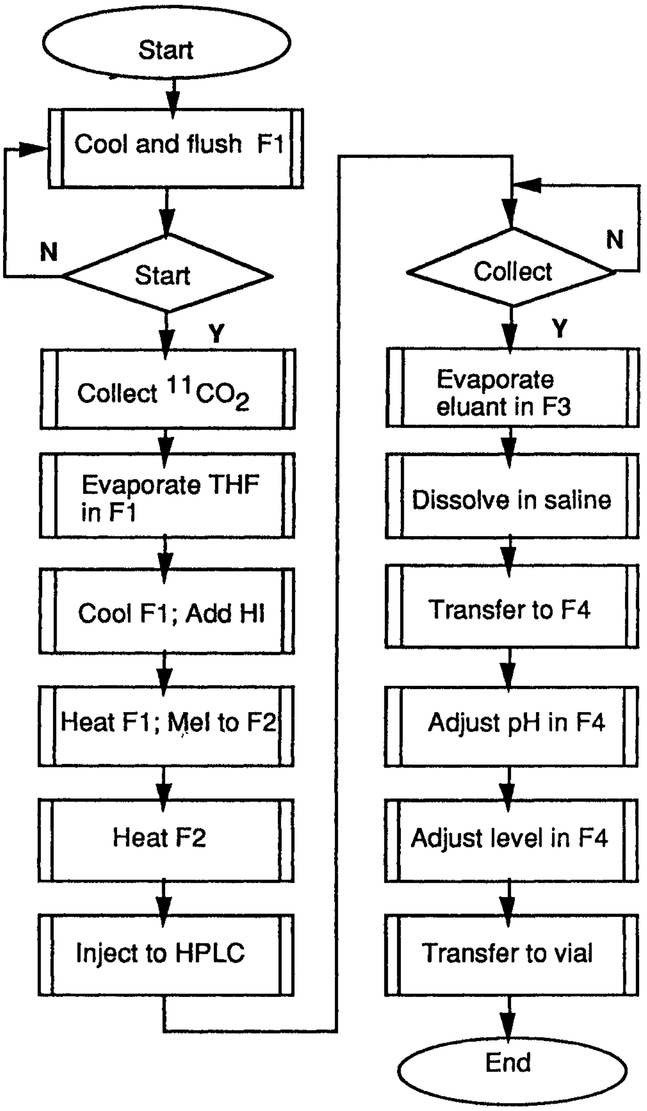

*Figure 6. Synthesis sequence (3-N-[$^{11}$C]methylspiperone).*

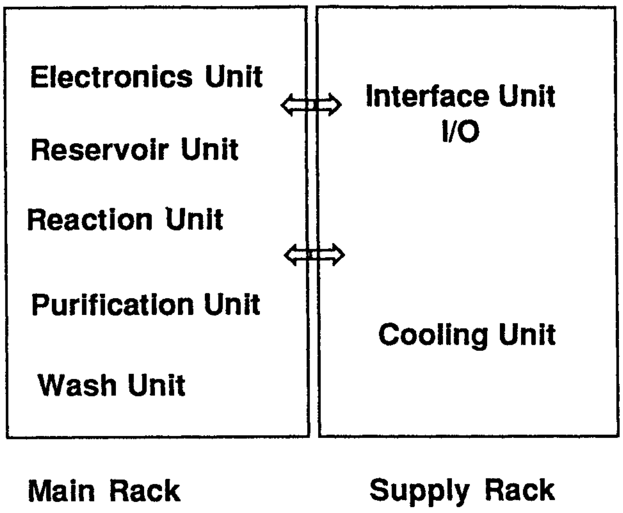

*Figure 7. Arrangement of the units on MIASA's two racks.*

*Reservoir supply unit*

The reagents, reactants and solvents that are to be used in the reaction flasks are stored in glass reservoirs, from which they are dispensed as required. Figure 11 shows the layout of the reservoir supply unit shelf.

The small volumes of liquids required, 100–200 μl from reservoirs R1 and R4, are measured out using fixed-position infra-red photosensors (EE-SX670, Omron) on the Teflon tube delivery lines. A block of black PVC was used to make a light shield which fits tightly into the well of the photosensor; holes for the Teflon tubing (1·6 or 3·0 mm ∅) and the light beam (1·0 mm ∅) cross in the centre of the light path. The basic circuit for control of dispensing from a reservoir is shown in figure 12. When a reservoir is opened (Va and Vb on) and the photosensor detects liquid, a signal is sent directly to the computer. Simultaneously, in order to achieve good reproducibility, the signal also switches a relay to immediately cut power to the solenoid valve at the bottom of the reservoir (Vc). A relay (S64) can switch off the operation of the photosensors to allow washing.

*Reaction unit*

The front of this unit contains four flasks on four stirrers. These are for:

(1) Synthesizing methyl iodide.
(2) Methylating the precursor compound.
(3) Collecting the separated product, evaporating eluant and dissolving in saline solution.
(4) Adjusting the pH and final concentration.

Flask F1 has an outer jacket for cooling and an inner jacket for heating. Coolant, fluorinert FC77, is circulated from a cold bath and heating is performed with a nichrome wire heater (*c.* 15 Ω) in silicone oil. The flask is designed so that all the tubing connections to the inner reaction vessel pass through the heating jacket; the flask is compact which helps the efficient removal of solvent from the connections during evaporation and drying processes.

Flask F2 has a single jacket containing fluorinert FC77, which can be heated by a nichrome wire heater (*c.* 20 Ω) or cooled by circulation from the cold bath. The four tubing connections pass through the jacket for compactness and efficient drying of the flask. Flask F3 has a single jacket filled with silicone oil, heated by a nichrome wire (*c.* 20 Ω). The magnetic stirring bar is supported on a Teflon disk on the Teflon tubing that dips down to the bottom of the flask. The tube is made rigid by an outer Teflon tube collar, so the stirrer bar spins freely. The Teflon tubing passes through a Teflon-coated silicone disk (septum) in the screw connector to minimize the dead space in the flask top (this is difficult to dry during evaporation). Flask F3 was also designed to minimize the loss of product through bumping. A glass protuberance is set just below the tubing connection to the vacuum line, blocking the direct loss of solution to the drain. Flask F4 is fitted with a small pH electrode (CE105-C, Nissin) and a level sensor to detect when the volume of the final solution reaches a pre-set value, for example 10 ml.

The rear of the reaction unit houses solenoid valves and a lead-shielded RI detector probe on the inlet line from the [$^{11}$C]carbon dioxide trap. A shelf above the reaction flasks holds a stepping motor (2CSM-101, Oriental

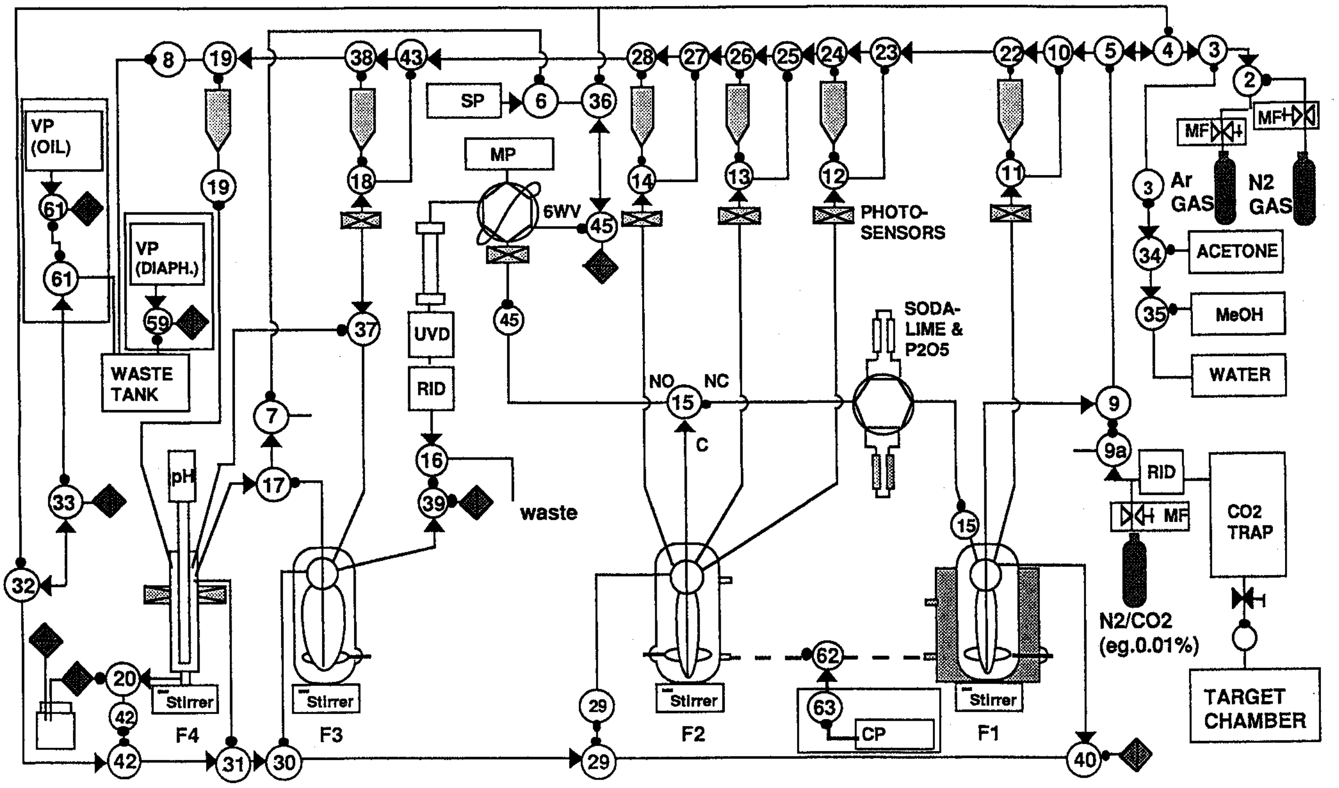

*Figure 8. Schematic diagram of the [$^{11}$C]MeI automated synthesis apparatus: ○ = solenoid valves; C = common; CP = cooler pump; MF = mass flow controller; NC = normally closed; NO = normally open; MP = mobile phase; pH = pH meter electrode; SP = syringe pump; RID = radioisotope detector; UVD = ultraviolet detector; VP = vacuum pump; 6WV = automated six-way injection valve.*

Motor) for operating a syringe pump (25 ml)—this pushes HPLC eluant from the injection loop to F2 and pulls the reaction solution back into the injection loop.

To ensure the reliability of the injection procedure, two photosensors are used for detecting the point when all the reaction solution has gone into the sample loop. When both photosensors go off, the sample is injected onto the HPLC column by a motor-operated six-way micro rotary valve (E010, Uniflows; 0·75 ml loop). Using two photosensors means that if a small air bubble were to pass through the Teflon tube, it would not accidentally cause premature injection. A third photosensor is fitted to the Teflon flow line just above F2 to detect when the push of eluant from the sample loop should be stopped. The set up shown in figure 13 was thus used to obtain reproducible injection.

A manual six-way injection valve (7125, Rheodyne; 0·6 ml loop), for calibration, and two manually operated six-way selection valves (7060, Rheodyne) for changing the HPLC flow line, and thus the HPLC column, are also located on the same shelf. Five columns and one bypass line can typically be connected.

When methyl iodide is passed from F1 to methylation flask F2 it is necessary to remove traces of excess acid (HI) and water by passing it through a trap containing soda lime and $P_2O_5$. The trap is fitted to a motor-operated six-way micro rotary valve (E010, Uniflows) to allow it to be by-passed during the washing and drying procedure, which can then follow immediately after synthesis. When the bypass is a second, empty, trap, it can be cleaned and made ready for use on the following run.

*Purification unit*

The two shelves below the reaction unit contain the purification apparatus, consisting of a compact HPLC pump (PU-980, Jasco) and UV detector (UV-970, Jasco), and a radioisotope detector (positron detector, Aloka). Figure 14 shows the layout of the unit.

*Vacuum, wash and drainage unit*

Also at the bottom of the main rack is a diaphragm type vacuum pump, a stainless-steel waste drain with a pressure sensor to allow monitoring of the vacuum, three polyethylene tanks for containing wash solvents and one tank to collect solvents from the vacuum pump exhaust line.

An oil-type vacuum pump is used to obtain sufficient suction for the evaporation in F3. A cooler, with a cold trap, condenses vapour before it enters the pump.

*Layout of the supply rack*

*OPTOMUX interface*

The top half of the supply rack contains the OPTOMUX I/O interface which connects the apparatus to the computer. The eight boards are housed in two boxes, together with the necessary power supplies.

*Digital I/O indicator and auto/manual switch box*

A digital I/O box contains 80 indicator lamps/switches for the five digital output boards and 16 indicator lamps

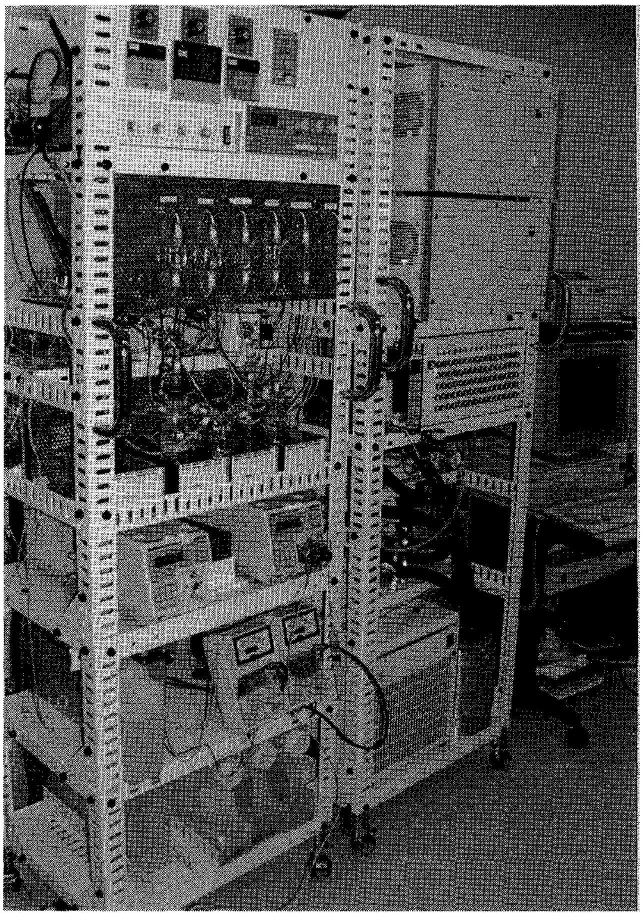

*Figure 9. General appearance of MIASA's two racks.*

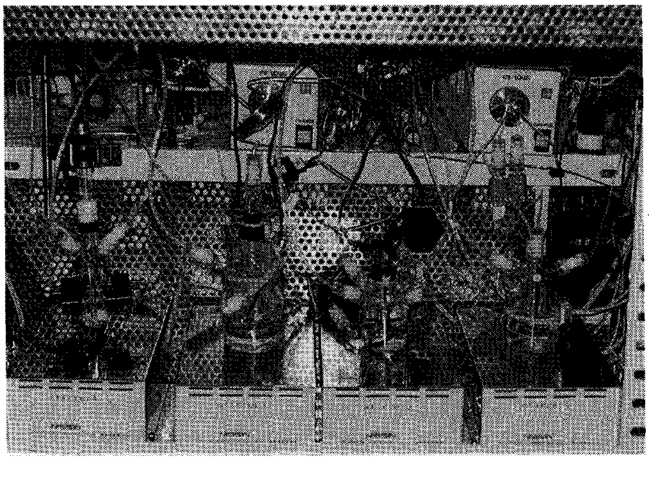

*Figure 10. MIASA's reaction unit.*

for the digital input board. One switch changes the apparatus control from auto (computer) to manual operation using the 80 switches. These switches simplify maintenance.

*Mass flow controllers*

Argon and nitrogen gas flow rates are monitored and regulated using mass flow controllers (0–500 ml/min;

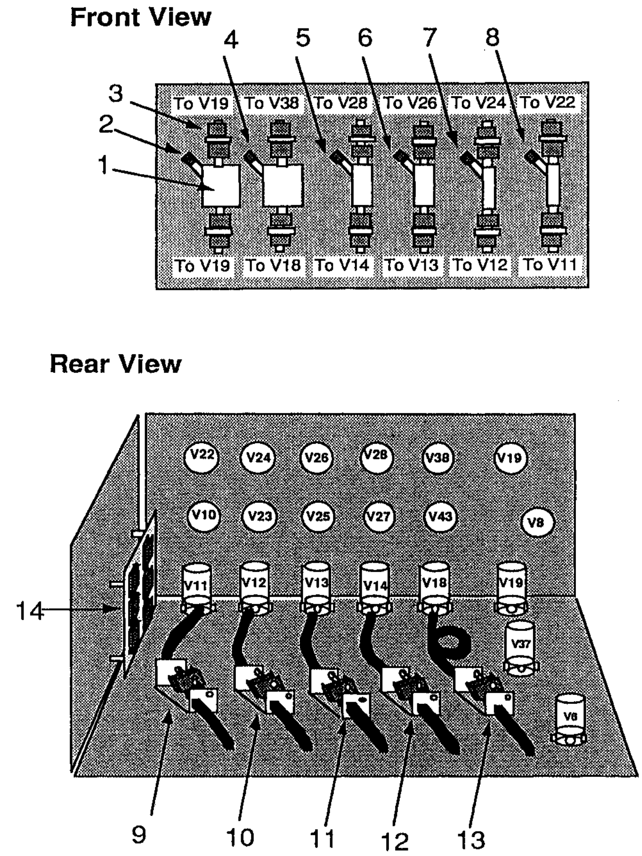

*Figure 11. The Reservoir Supply Unit: 1 = glass reservoir (acid or base for pH adjustment); 2 = silicon septum; 3 = teflon connector (5–3 mm ∅); 4 = saline reservoir; 5–7 = reservoirs for F2 reactants; 8 = HI acid reservoir; 9–13 = photosensors; 14 = photosensor local control relay board.*

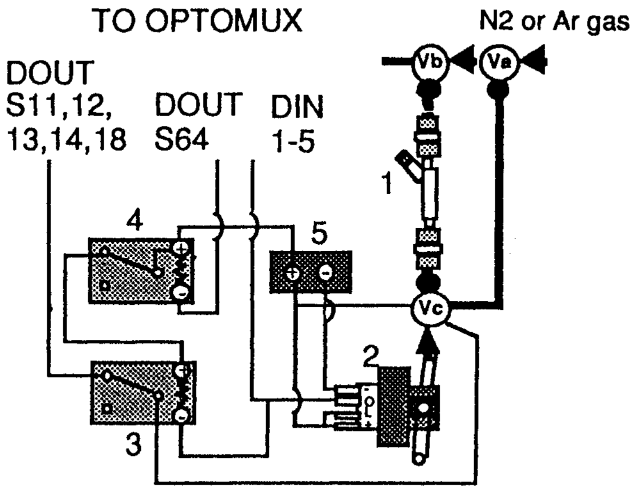

| | Va | Vb | Vc |
|---|---|---|---|
| Measure | OFF | ON | ON |
| Dispense | ON | OFF | OFF |

*Figure 12. Basic circuit for dispensing from reservoirs. 1 = reservoir; 2 = photosensor; 3 = 24 V DC relay switching Vc off; 4 = 24 V DC relay switching 24 V power off; 5 = 24 V DC power supply.*

*Table 1. I/O connections of the MIASA apparatus.*

| Board | Module | Pin # | Description | Switch # |
|---|---|---|---|---|
| Digital output (DOUT) | ODC5 | 1 | Not connected | V1 |
| Address 00H | ODC5 | 2 | Valve 2, connect $N_2$ gas line to reservoir line | V2 |
| | ODC5 | 3 | Valve 3, connect wash line to reservoir line | V3 |
| | ODC5 | 4 | Valve 4, connect reservoir line to drain line | V4 |
| | ODC5 | 5 | Valve 5, connect reservoir line to F1 | V5 |
| | ODC5 | 6 | Valve 6, connect syringe pump to F2 | V6 |
| | ODC5 | 7 | Valve 7, connect syringe pump to F4 | V7 |
| | ODCS | 8 | Valve 8, connect reservoir line to drain | V8 |
| | ODC5 | 9 | Valve 9, connect [$^{11}$C]$CO_2$ inlet line to F1 | V9 |
| | ODC5 | 10 | Valve 10, connect reservoir line to F1 | V10 |
| | ODC5 | 11 | Valve 11, connect reservoir #1 to F1 | V11 |
| | ODC5 | 12 | Valve 12, connect reservoir #2 to F2 | V12 |
| | ODC5 | 13 | Valve 13, connect reservoir #3 to F2 | V13 |
| | ODC5 | 14 | Valve 14, connect reservoir #4 to F2 | V14 |
| | ODC5 | 15 | Valve 15, connect F1 to F2 | V15 |
| | ODC5 | 16 | Valve 16, connect HPLC line to F3 | V16 |
| Address 01H | ODC5 | 17 | Valve 17, connect F3 to F4 | V17 |
| | ODC5 | 18 | Valve 18, connect reservoir #5 to F3 or F4 | V18 |
| | ODC5 | 19 | Valve 19, connect reservoir #6 to F4 | V19 |
| | ODC5 | 20 | Valve 20, connect F4 to vial | V20 |
| | ODC5 | 21 | Valve 21, connect F2 outlet to balloon | V21 |
| | ODC5 | 22 | Valve 22, connect reservoir line to res #1 | V22 |
| | ODC5 | 23 | Valve 23, connect reservoir line to F2 | V23 |
| | ODC5 | 24 | Valve 24, connect reservoir line to res #2 | V24 |
| | ODC5 | 25 | Valve 25, connect reservoir line to F2 | V25 |
| | ODC5 | 26 | Valve 26, connect reservoir line to res #3 | V26 |
| | ODC5 | 27 | Valve 27, connect reservoir line to F2 | V27 |
| | ODC5 | 28 | Valve 28, connect reservoir line to res #4 | V28 |
| | ODC5 | 29 | Valve 29, connect F2 to drain line | V29 |
| | ODC5 | 30 | Valve 30, connect F3 to drain line | V30 |
| | ODC5 | 31 | Valve 31, connect F4 to drain line | V31 |
| | ODC5 | 32 | Valve 32, connect drain line to V4 and V36 | V32 |
| Digital output (DOUT) | ODC5 | 33 | Valve 33, connect drain line to vacuum pump | V33 |
| Address 02H | ODC5 | 34 | Valve 34, connect acetone tank to wash line | V34 |
| | ODC5 | 35 | Valve 35, connect methanol tank to wash line | V35 |
| | ODC5 | 36 | Valve 36, connect HPLC loop to drain line | V36 |
| | ODC5 | 37 | Valve 37, connect reservoir #5 to F4 | V37 |
| | ODC5 | 38 | Valve 38, connect reservoir line to res #5 | V38 |
| | ODC5 | 39 | Valve 39, connect HPLC delivery line to F3 | V39 |
| | ODC5 | 40 | Valve 40, connect F1 to soda lime trap | V40 |
| | ODC5 | 41 | Not connected | V41 |
| | ODC5 | 42 | Valve 42, connect F4 to drain | V42 |
| | ODC5 | 43 | Valve 43, connect reservoir line to F3 | V43 |
| | ODC5 | 44 | Relay 44, switch off HPLC pump | S44 |
| | ODC5 | 45 | Valve 45, connect HPLC loop to F2 & s. pump | V45 |
| | ODC5 | 46 | Relay 46, start HPLC pump | S46 |
| | ODC5 | 47 | Relay 47, change position of 6WV: SLT/$P_2O_5$ | V47 |
| | ODC5 | 48 | Relay 48, change position of 6WV: HPLC loop | V48 |
| Address 03H | ODC5 | 49 | Relay 49, switch heater #1 on | S49 |
| | ODC5 | 50 | Relay 50, switch heater #2 off | S50 |
| | ODC5 | 51 | Relay 51, switch heater #3 on | S51 |
| | ODC5 | 52 | Relay 52, switch stirrer #1 off | S52 |
| | ODC5 | 53 | Relay 53, switch stirrer #2 off | S53 |
| | ODC5 | 54 | Relay 54, switch stirrer #3 off | S54 |
| | ODC5 | 55 | Relay 55, switch stirrer #4 off | S55 |
| | ODC5 | 56 | Relay 56, start syringe pump | S56 |
| | ODC5 | 57 | Relay 57, set syringe pump to pull | S57 |
| | ODC5 | 58 | Relay 58, set UV auto zero | S58 |
| | ODC5 | 59 | Relay 59, switch diaphragm pump on | S59 |
| | ODC5 | 60 | Relay 60, switch UV detector on | S60 |
| | ODC5 | 61 | Relay 61, switch vacuum pump (oil) on | S61 |

*Table 1* (*continued*)

| Board | Module | Pin # | Description | Switch |
|---|---|---|---|---|
| Address 03H | ODC5 | 62 | Relay 62, switch valve 62 on—coolant to F2 | S62 |
| | ODC5 | 63 | Relay 63, switch cooling pump on | S63 |
| | ODC5 | 64 | Relay 64, switch photosensor power off | S64 |
| Digital input (DIN) | IDC5 | DIN1 | Photosensor #1 for reservoir #1 | |
| Address 05H | IDC5 | DIN2 | Photosensor #2 for reservoir #2 | |
| | IDC5 | DIN3 | Photosensor #3 for reservoir #3 | |
| | IDC5 | DIN4 | Photosensor #4 for reservoir #4 | |
| | IDC5 | DIN5 | Photosensor #5 for reservoir #5 | |
| | IDC5 | DIN6 | Photosensor #6 for HPLC injection | |
| | IDC5 | DIN7 | Level sensor on F4 | |
| | IDC5 | DIN8 | Position of six-way valve for HPLC injection | |
| | IDCS | DIN9 | Position of six-way valve for HPLC SLT/$P_2O_5$ | |
| | IDC5 | DIN10 | Position of syringe pump | |
| | IDC5 | DIN11 | Photosensor #11 above F2 | |
| | IDC5 | DIN12 | Not connected | |
| | IDC5 | DIN13 | Not connected | |
| | IDC5 | DIN14 | Not connected | |
| | IDC5 | DIN15 | Not connected | |
| | IDC5 | DIN16 | Not connected | |
| Analogue input (AIN) | AD6T | AIN1 | Not connected | |
| Address 06H | AD6T | AIN2 | pH meter (0–5 V) | |
| | AD6T | AIN3 | Vacuum gauge (0–5 V) | |
| | AD6T | AIN4 | Reading mass flow controller (0–5 V) | |
| | AD6T | AIN5 | Not connected | |
| | AD6T | AIN6 | Not connected | |
| | AD9T | AIN7 | UV detector (0–50 mV) | |
| | AD9T | AIN8 | RI detector (0–50 mV) | |
| | AD9T | AIN9 | Not connected | |
| | AD8T | AIN10 | K type thermocouple for F1 | |
| | AD8T | AIN11 | K type thermocouple for F3 | |
| | AD8T | AIN12 | K type thermocouple for cold bath | |
| | AD3T | AIN13 | Not connected | |
| | AD3T | AIN14 | Reading F2 temperature controller (4–20 mA) | |
| | — | AIN15 | Not connected | |
| | – | AIN16 | Not connected | |
| Analogue output (AOUT) | DA3T | AOUT1 | Setting F2 temperature comtroller (4–20 mA) | |
| Address 07H | DA3T | AOUT2 | Not connected | |
| | DA4T | AOUT3 | Not connected | |
| | DA4T | AOUT4 | Setting mass flow controller (0–5 V) | |

valve SEC-400, control PAC-S5 v2, STEC Japan). These are used to maintain consistent synthesis and to search for leaks during the diagnostic check before synthesis. They are important for maintaining the reliability of the apparatus.

### *AC 100 V supply*

A series of AC 100 V outlets with safety breakers supply power to both racks.

### *Cooling system*

The lower half of the supply rack contains the cooling system, consisting of a 'cool pipe' cooler with a minimum temperature of $-50°C$, a circulating micro pump, a voltage controller and a 3 l Dewar tank of coolant (fluorinert FC77). Solenoid valves are used to stop and direct the flow of coolant to F1 or F2.

The software was developed to be compatible with the total automated-labelling production system, including the cyclotron and the PET camera. MIASA reliably completes the link between the production of the positron emitter radionuclide, $^{11}C$, and the delivery of the $^{11}C$-labelled radiopharmaceutical for PET study.

### Application to the synthesis of 3-*N*-[$^{11}$C]methylspiperone

Spiperone, 1, and its *N*-methylated analogue, 2, are widely used butyrophenone neuroleptics that have been labelled with short-half life positron emitters, such as $^{11}C$ ($t_{1/2} = 20{\cdot}4$ min) and $^{18}F$ ($t_{1/2} = 109{\cdot}6$ min), to give useful ligands for studying both dopamine and serotonin receptor binding *in vivo* [7, 8]. The synthesis of

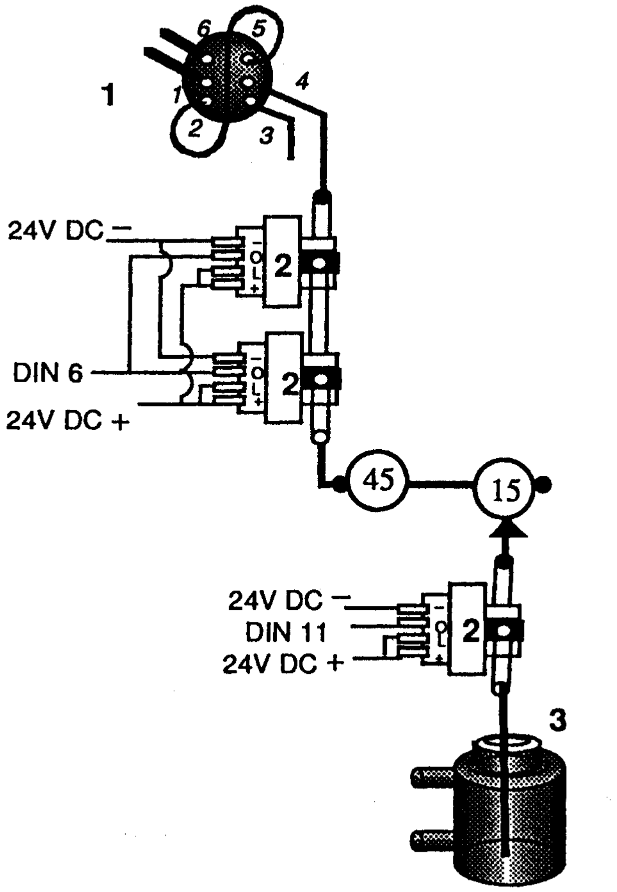

*Figure 13. Set up of the sensors for Controlling HPLC Injection. 1 = 6-way micro-rotary valve for HPLC auto-injection; 2 = photosensors; 3 = flask #2.*

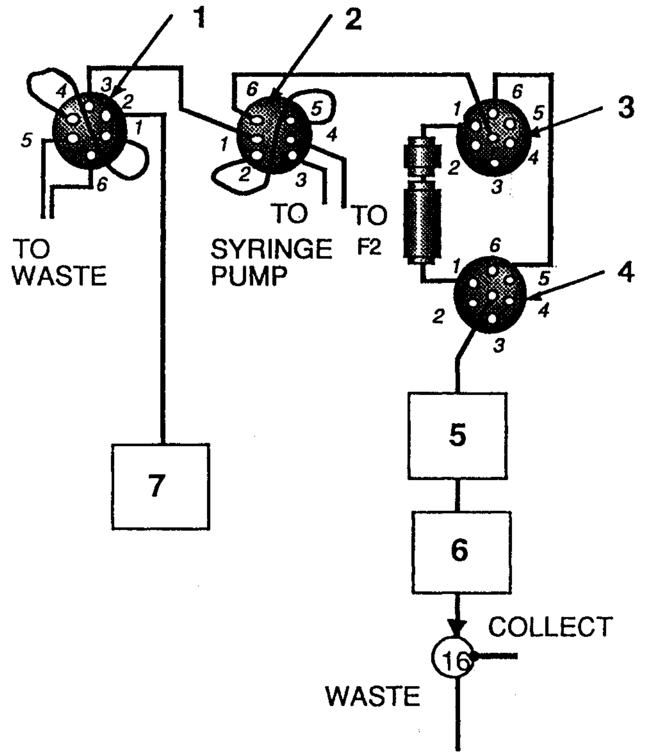

*Figure 14. Layout of the Purification Unit. 1 = HPLC injection valve for manual use; 2 = 6-way micro-rotary valve for HPLC auto-injection; 3,4 = 6-way selection valves; 5 = HPLC UV detector; 6 = positron detector; 7 = HPLC pump.*

3-*N*-[$^{11}$C]methylspiperone was used to test the operation of the system.

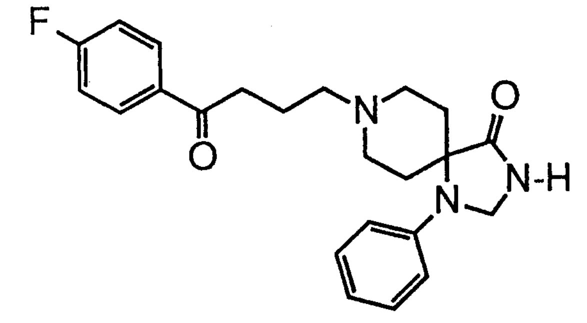

1

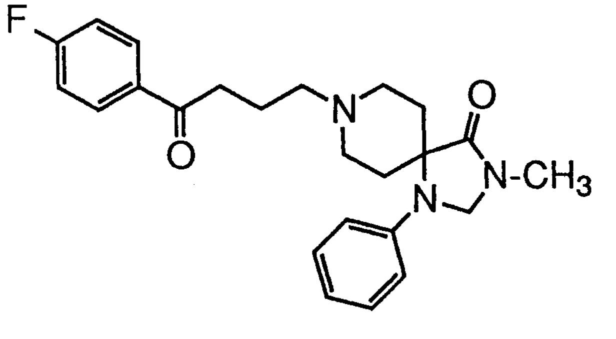

2

### Experiments

### *Materials and reagents*

Materials and reagents were purchased as follows: spiperone and 2-*N*-methylspiperone from Janssen Pharmaceuticals or Funakoshi Pharmaceuticals; Tetrahydrofuran (THF), lithium aluminium hydride (LAH) powder, tetrabutylammonium hydroxide 10% aq. (TBAOH), hydroiodic acid 57% aq., 1,2-dichlorobenzene and dichloromethane were from Wako Chemicals Ltd; 1·0 M solution of LAH/THF was from Aldrich Chemicals Ltd; sodalime (grade No. 1) was from Kanto Chemicals; and phosphorus pentoxide from Fluka Chemie AG. Isotonic saline solution was from Otsuka Pharmaceuticals. The 0·22 μm sterilizing filter was from Milex (GV25 for low adsorption).

### *Preparation of the apparatus*

THF was freshly distilled over lithium aluminium hydride under argon atmosphere. After refluxing for 2–3 h the distillate was collected in a trap, and a portion (18 ml) withdrawn through a rubber septum using a gas-tight syringe. The THF was injected into a vial (30 ml), capped with a Teflon-lined septum and flushed with argon. Addition of 2·0 ml of a commercial 1·0 M LAH/THF solution gave a 0·1 M solution of LAH/THF that was stored in a desiccator over $P_2O_5$ and silica gel until use.

The reservoirs of the apparatus were filled as follows:

Reservoir #1 (R1)—c. 0·5 ml of HI(aq) (57%).
Reservoir #2 (R2)—c. 0·5 ml of a spiperone solution in a 70:30 v/v mixture of 1,2-dichlorobenzene and dichloromethane (0·83 × $10^{-3}$ M).
Reservoir #3 (R3)—c. 0·5 ml of TBAOH(aq) (10%).
Reservoir #4 (R4)—c. 0·5 ml of a 56:44 v/v mixture of 1 M HCl(aq)/THF (0·56 M).
Reservoir #5 (R5)—c. 10 ml isotonic saline/ethanol (100:5 v/v).
Reservoir #6 (R6)—c. 5 ml $Na_2CO_3$(aq) (0·1 M).

The $P_2O_5$/sodalime trap was filled and the six-way valve holding the trap was set to direct the flow through the trap. The drying tube on the argon line was filled with 2–3 g of $P_2O_5$. The HPLC eluant was prepared by mixing disodium hydrogen citrate (0·04 M) and methanol in the ratio 52·5:47·5 v/v, followed by degassing under reduced pressure and sonication. An authentic sample of 3-*N*-methylspiperone in the eluant was manually injected (c. 500 μl; 50 nmol), and the retention time confirmed to be about 10 min using the following chromatographic conditions: column, Capcell pak SG120 (15 × 150 mm + 15 × 30 mm precolumn); flow rate, 9·5 ml/min; wavelength, 254 nm.

*Diagnostic check of the apparatus*

The status of the apparatus was checked to be ready for synthesis, as outlined in figure 5. The position of the six-way rotary valves was checked and set to the correct starting position if necessary. Similarly, the position of the syringe pump was checked and photosensors on the reservoir lines were confirmed to be off. Leak tests on F1–F4 and the sodalime/$P_2O_5$ trap were performed by closing all outlets, opening them to the argon flow line and monitoring the mass flow controller reading. If zero flow could not be obtained, the source of the leak was searched for and remedied.

*Get ready to start a synthesis*

Flask F1 was cooled to about −20°C (S63 on) and flushed with dry argon gas (V5 on) before the LAH/THF solution (100 μl, 10 μmol LAH) was injected. Cooling was continued while the target was irradiated for up to 40 min.

*Collection of [$^{11}$C]carbon dioxide*

The contents of the target were swept to the cryotrap and [$^{11}$C]carbon dioxide was trapped in a coiled tube dipping into liquid argon. The start of transfer, SOT, to MIASA was begun by raising the coil from the liquid argon and flushing with He gas (10 ml/min). The radioisotope detector on the inlet line was used to monitor the release of radioactivity from the trap, and valves V9/V9a were opened to direct the flow to F1 when the peak of $^{11}CO_2$ was detected. The outlet from F1 (V40) was opened to a sodalime trap to prevent any leakage of radioactive gas. The peak of radioactivity was collected for 2–3·5 min for a 20–40 min irradiation of the target ($N_2$: 14·7 kg/cm$^2$; 15 μA).

During the collection of $^{11}CO_2$, spiperone solution was added to F2. The outlet of F2 was opened by switching on V29 and measurement from R2 was performed by opening V12 and V24 until photosensor #2 (PS2) switched on, and then opening V23 to flush the measured volume to F2. Addition was repeated three times to add a total of 300 μl, 1 mg spiperone.

*[$^{11}$C]Methyl iodide synthesis and labelling*

After the collection of $^{11}CO_2$ in F1 the THF was evaporated by evacuation and heating to 130°C for 2 min. Flask F1 was then cooled to c. 45°C by switching on the cooling pump. Valves V15 and V29 were opened and HI(aq) solution (150 μl) was added from R1. This addition immediately released [$^{11}$C]methanol, which was then converted to [$^{11}$C]methyl iodide by reaction with HI(aq) solution on further heating to 130°C. [$^{11}$C]Methyl iodide was transferred to F2 under a stream of argon gas at 10 ml/min, through the sodalime/$P_2O_5$ trap. Flask F2 was cooled for a short period before transfer, to improve the trapping efficiency of [$^{11}$C]methyl iodide in the spiperone solution. In order to determine how much [$^{11}$C]methyl iodide was not trapped in F2, the outlet gas was directed (V21 on) into a balloon put inside a Curie meter.

Transfer from F1 was stopped after 3 min by closing V15, and TBAOH base (200 μl) was added to F2 from R3. Flask F2 was completely closed and then heated to 65°C, set with the temperature controller (AOUT1). The HPLC pump was also started in preparation for the next step.

*HPLC injection*

After 4 min reaction, the injection of the reaction mixture into the HPLC column was started. The six-way micro rotary valve, holding the injection loop, was turned to the load position and valves V45 and V29 were opened. The eluant in the loop was pushed by the syringe pump to fill the Teflon tube between the loop and F2. When photosensor #11 (PS11) detected the liquid just above F2, a short time (3 s) was allowed for the tubing to be completely filled, and then the push was stopped by closing V45. The reaction mixture in F2 was neutralized by addition of dilute HClaq (1 M)/THF (56:44 v/v) from R4. Valves V14 and V28 were opened until photosensor #4 (PS4) switched on, and then V27 was opened to flush the measured volume of acid (100 μl) to F2. Stirring in F2 was stopped and the two-phase reaction mixture allowed to separate. The addition of THF with the acid helped to speed up the separation and reduce the cloudiness of the mixture, which was essential for the operation of the photosensors. The reaction mixture in F2 was loaded into the injection loop by opening V45 and pulling with the syringe pump until photosensor #6 (PS6) detected that no solution remained in the Teflon tube. The HPLC six-way valve was then turned to inject. The heater of F3 was then switched in preparation for the subsequent evaporation step.

*Product collection*

The UV and radioisotope detectors on the HPLC line were monitored and recorded on the computer, and the

3-*N*-[$^{11}$C]methylspiperone peak eluting at *c.* 10 min was collected into F3 by opening V16. Nitrogen gas was bubbled through F3 to start the evaporation of the eluant as soon as collection proceeded. Collection was stopped by closing V16, and evaporation was continued under evacuation. Opening V39 at the same time ensured that the delivery line into F3 was emptied and also helped to prevent loss of product by bumping.

In order to determine the radiochemical yield and specific activity at end of synthesis (EOS), the product could be collected into a volumetric flask placed in a Curie meter (runs HR6, HR8 and HR9), and no further formulation was performed. The collection fraction was made up to 25 ml with HPLC mobile phase after radioactivity had decayed and the amount of 3-*N*-methylspiperone was analyzed by HPLC.

### *Product formulation*

After allowing 9 min for evaporation to dryness in F3, the heater and vacuum pump were switched off and saline solution (5 ml) added from R3. The product was dissolved in the saline by stirring and bubbling nitrogen gas. Applying the vacuum pump for a short time (2 s) improved the dissolution of the product by making the bubbling of nitrogen gas more vigorous, which resulted in more effective washing of the top part of F3 with the saline solution.

Flask F4 was emptied of water by opening valves V42, V33 and switching on the vacuum pump, and then the saline solution was transferred from F3 for pH adjustment. Sodium carbonate solution (0·1 M) was added from R6 by repeatedly opening V19 for 1 s, until the pH was within the range 7·0 $\pm$ 1·0. The volume of the final solution in F4 was adjusted to 10 $\pm$ 1 ml (set by the level sensor position) by adding more saline from R5. Valves V18 and V38 were opened for 1 s, and then V43 and V37 were opened to flush saline to F4.

Finally, the product solution was filtered through a 0·22 μm sterile filter into a sealed vial by applying nitrogen gas pressure. The vial was placed in a Curie meter to allow the amount of radioactivity in the product to be measured. The specific activity of the product was calculated after HPLC analysis of the 3-*N*-methylspiperone in the final solution at 18 h after end of bombardment (EOB).

### *Washing*

Washing of the apparatus could be started immediately after collection of the product. The position of the six-way valve holding the sodalime/$P_2O_5$ trap was changed to direct the flow through a bypass and the photosensors on the reservoir lines were switched off with a relay. F1 and R1 were washed with water and then the soda lime/$P_2O_5$ trap and F2 were washed by opening V15 and V29 to pass water from F1. F2 was also washed from the reservoir line via each of V23, V25 and V27. Washing was then repeated with acetone. F3 and F4 were washed with methanol and water, leaving water in F4 to preserve the pH meter.

The HPLC column was washed with water (*c*, 3 min, 9·5 ml/min), 90% methanol (*c.* 12 min, 9·5 ml/min) and then water again (*c.* 3 min, 9·5 ml/min), to remove dichlorobenzene and prevent build up of impurities.

### Results and discussion

The total synthesis time from the end of bombardment to production of the sterilized saline solution was approximately 40 min. Table 2 shows the amounts and specific activities of 3-*N*-[$^{11}$C]methylspiperone produced on several runs, including runs where the product was collected before formulation. For a 40 min irradiation using 18 MeV protons at 15 μA, producing *c.* 1 Ci of [$^{11}$C]$CO_2$, the specific activity was over 1·5 Ci/μmol at the end of synthesis and *c.* 1 Ci/μmol at the end of formulation. The decay corrected radiochemical yield of the 3-*N*-[$^{11}$C]methylspiperone from [$^{11}$C]$CO_2$, in the injectable solution, was approximately 25%. The losses of

*Table 2. Results of synthetic runs for 3-N-[$^{11}$C]methylspiperone (NMSP).*

| Run no. | Target irradiation min/μA/kgf cm$^{-2}$ | NMSP at EOS[a] nmol | NMSP at EOF[b] nmol | RI at EOS[c] mCi | RI at EOF[d] mCi | Sp. Act. at EOS[e] mCi/μmol | Sp. Act. at EOF[f] mCi/μmol | Approx. RCY[g] % |
|---|---|---|---|---|---|---|---|---|
| HR6 | 20/15/14·7[h] | 117 | — | 149·6 | — | 1279 | — | 83 |
| HR8 | 20/15/14·7[j] | 59 | — | 71 | — | 1203 | — | 31 |
| HR9 | 40/15/14·7[k] | 66 | — | 112·5 | — | 1705 | — | 31 |
| HR10 | 40/15/14·7[k] | 85 | 64 | 136[m] | 72·9[n] | 1600 | — | 38 |
| HR11 | 40/15/14·7[k] | 82 | 54 | 146[m] | 58·3 | 1800 | 1080 | 25 |

*a.* Calculated from UV detector peak area at separation by HPLC (end of synthesis).
*b.* Calculated from UV detector peak area on HPLC analysis of formulated product (18 h after end of bombardment).
*c.* Measured by collecting product from HPLC into a flask placed inside a Ci meter.
*d.* Measured by collecting product from F4 into a vial placed inside a Ci meter.
*e.* Calculated specific activity of product collected after HPLC separation.
*f.* Calculated specific activity of sterile product collected in vial.
*g.* Estimated decay corrected radiochemical yields based on expected amount of [$^{11}$C]carbon dioxide produced.
*h.* 30 min required, as beam current lost for 10 min, *c.* 450 mCi [$^{11}$C]$CO_2$ expected.
*j.* *c.* 580 mCi [$^{11}$C]$CO_2$ expected.
*k.* *c.* 946 mCi [$^{11}$C]$CO_2$ expected.
*m.* Estimated radioactivity from measured values at EOF.
*n.* Includes radioactivity retained on sterilizing filter.

radioactivity that were measured (Runs HR10 and HR11) included 0·5% as [$^{11}$C]$CO_2$ and 12% as [$^{11}$C]-methyl iodide (decay corrected radiochemical yields).
Thus the synthesis and formulation of 3-*N*-[$^{11}$C]methyl-spiperone with the fully automated apparatus MIASA was sufficiently fast for a high specific activity product to be obtained, suitable for dopamine and serotonin receptor binding studies. The specific activity previously reported by Burns *et al.* [9], using a similar reaction condition, was significantly lower (270 mCi/μmol). The high specific activity is presumably a result of the precautions taken in the preparation and handling of [$^{11}$C]methyl iodide— notably in the preparation of the LAH/THF solution, the conditioning of the flow lines and F1 with dry argon gas before synthesis and the use of high purity (99·9999%) nitrogen gas as the target material.
Improvements were made to the hardware and software during the development of the apparatus. For example the use of a mass flow controller, to monitor and regulate gas flows, aided the detection and elimination of leaks prior to synthesis runs. Safety and reliability were improved. The use of three photosensors to control the injection procedure, improved the reproducibility of this crucial step.

The computer interface unit (OPTOMUX) and software are compatible with the total production system of PET radiopharmaceuticals. In future, it will be possible (using the present hardware) to use feedback to monitor and control such processes as solvent evaporation (THF in F1) and temperature maintenance.

3-*N*-[$^{11}$C]Methylspiperone was synthesized to show the performance of the apparatus; MIASA can easily be adapted to synthesize a variety of [$^{11}$C]methylated compounds. The selective dopamine receptor ligand, [$^{11}$C]raclopride, has also been synthesized.

### Acknowledgements

This research work was partly supported by a grant from the Japan Health Science Foundation. The authors also wish to acknowledge Dr Teruo Omae, President of the National Cardiovascular Center, for supporting this work.

### References

1. Vandewalle, T., Vandecasteele, C., De Guchteneire, F., Meulewaeter, L., van Haver, D., Denutte, H., Goethals, P. and Slegers, G., *International Journal of Applied Radiation and Isotopes*, **36** (1985), 469.
2. Alexoff, D. L., Russell, J. A. G., Shiue, C. Y., Wolf, A. P., Fowler, J. S. and MacGregor, R. R., *International Journal of Applied Radiation and Isotopes*, **37** (1986), 1045.
3. Takashashi, T., Ido, T., Iwata, R., Hatano, K., Nakanishi, H., Shinohara, M. and Shigenori, I., *International Journal of Applied Radiation and Isotopes*, **39** (1988), 659.
4. Hamacher, K., Blessing, G. and Nebeling, B., *International Journal of Applied Radiation and Isotopes*, **40** (1989), 49.
5. Padgett, H. C., Schmidt, D. G., Luxen, A., Bida, G. T., Satyamurthy, N. and Barrio, J. R., *International Journal of Applied Radiation and Isotopes*, **40** (1989), 433.
6. Adam, M. J. and Jivan, S., *International Journal of Applied Radiation and Isotopes*, **39** (1988), 1203.
7. Maziere, B., Coenen, H. H., Halldin, C., Nagren, K. and Pike, V. W., *Nuclear Medicine and Biology*, **19** (1992), 497.
8. Wong, D. F., Wagner Jr., H. N., Tune, L. E., Dannals, R. F., Pearlson, G. D., Links, J. M., Tamminga, C. A., Broussolle, E. P., Ravert, H. T., Wilson, A. A., Toung, J. K. T., Malat, J., Williams, J. A., O'Tuama, L. A., Snyder, S. H., Kuhar, M. J. and Gjedde, A., *Science*, **234** (1986), 1558.
9. Burns, H. D., Dannals, R. F., Langström, B., Ravert, H. T., Zemyan, S. E., Duelfer, T., Wong, D. F., Frost, J. J., Kuhar, M. J. and Wagner Jr., H. N., *Journal of Nuclear Medicine*, **25** (1984), 1222.

